# Changes in energy homeostasis, gut peptides, and gut microbiota in Emiratis with obesity after bariatric surgery

**DOI:** 10.1371/journal.pone.0318699

**Published:** 2025-02-24

**Authors:** Manal Ali Ahmad, Koen Venema, Carole Ayoub Moubareck, Gabi Wazz, Tarek Mahdy, Mirey Karavetian

**Affiliations:** 1 School of Nutrition and Translational Research in Metabolism (NUTRIM), Faculty of Health, Medicine and Life Sciences, Maastricht University, Maastricht, The Netherlands; 2 Wageningen Food and Biobased Research, Wageningen University and Research, Wageningen, The Netherlands; 3 College of Natural and Health Sciences, Zayed University, Dubai, United Arab Emirates; 4 Center of Excellence in Bariatric and Metabolic Surgery, Dr. Sulaiman Al Habib Hospital, Dubai, United Arab Emirates; 5 Department of Bariatric Surgery, Sharjah University, Sharjah, United Arab Emirates; 6 Faculty of Kinesiology and Physical Education, University of Toronto, Toronto, Ontario, Canada; National Healthcare Group, SINGAPORE

## Abstract

**Background:**

Obesity is a growing health concern worldwide, including United Arab Emirates. Bariatric surgery is an effective treatment option, with to date unclear weight loss mechanisms. In this prospective study, we explored post-bariatric surgery changes in energy homeostasis, gut peptides, hormones, and gut microbiota.

**Method:**

We recruited 19 Emirati adults who were planning to undergo sleeve gastrectomy (SG). We assessed the energy requirements using 24-hour diet recalls, indirect calorimetry for resting energy expenditure (REE), and a questionnaire for appetite.

Anthropometrics included body mass index (BMI), waist circumference, waist-to-height ratio, fat mass, fat-free mass, and percentage of body fat. Gut peptides, including peptide YY (PYY), glucagon-like peptide-1/2 (GLP-1/2), ghrelin (GHR), cholecystokinin (CCK), insulin, and leptin, were quantified using ELISA. Gut microbiota composition at phylum and genus levels, including the Firmicutes/Bacteroidetes (F/B) ratio and alpha (*α*) and beta (*β*) diversity, was determined by sequencing amplicons of the V3-V4 region of the 16S rRNA at baseline and three months post-surgery. Comparisons used paired sample T-test, Wilcoxon, and McNemar test. QIIME 2 was used to identify taxa and their relative abundance; subsequent analyses were done in R for (*α*) and (*β*) diversity (package qiime2R) and Wilcoxon signed-rank test in R for differences in microbiota at phylum and genus levels. We conducted Spearman correlation analyses between genera and energy homeostasis, appetite, anthropometrics, hormones, and gut peptides.

**Results:**

At three months post-SG, energy intake, appetite, all anthropometric indices, insulin, leptin, and GLP-1 significantly decreased; PYY and GHR significantly increased, and REE was stable. β-diversity of the gut microbiota and its composition at phylum and genus levels significantly changed post-surgery, yet F/B remained constant. Energy intake, BMI, and appetite negatively correlated with several taxa that significantly increased post-SG.

**Conclusion:**

Gut peptides, hormones, and microbiota change partly account for bariatric surgery’s weight-loss benefits. Understanding these alterations can inform personalized interventions targeting obesity.

## Introduction

Obesity rates have dramatically increased over the last two decades [1]. By 2023, it is anticipated that more than one billion individuals globally will be affected by this trend [2]. The situation is not different in the Arabian Gulf countries such as the United Arab Emirates (UAE), where obesity prevalence is believed to have at least doubled between 1989 and 2017 [3], reaching 24% among males and 40% among females [4].

In addition to diet, lifestyle, and genetics, gut microbiota has surfaced as a potential factor influencing obesity and related metabolic disturbances [5,6]. Proposed mechanisms involve the gut microbiota’s impact on gut peptides, appetite, energy balance, and fat storage [7–12] through a bidirectional signaling axis known as the “microbiota-gut-brain axis” [10,13].

Given the rising prevalence of obesity and the challenges of adhering to lifestyle, dietary, and pharmacotherapy interventions [14], bariatric surgery has gained recognition as an effective long-term adjunct for managing obesity and its associated comorbidities [15,16]. Bariatric surgery can be restrictive or malabsorptive [17]. Sleeve gastrectomy (SG) is a restrictive surgery. The precise mechanisms responsible for weight loss and metabolic alterations associated with SG have not been entirely clarified. Nonetheless, potential factors that may play a role include changes in food preferences, decreased food intake, heightened satiety, improved gastric emptying, reduced nutrient absorption, and increased energy expenditure [18–22]. These mechanisms may, in part, be a result of post-bariatric surgery alterations in gut peptides such as glucagon-like peptide 1 (GLP-1) and peptide YY (PYY) [23–27], and, as mentioned above, these could be influenced by the gut microbiota. Recent findings indicate a substantial modification in the composition and diversity of the gut microbiota after bariatric surgery. Key reported changes encompass increased levels of Proteobacteria [new nomenclature Pseudomonadota] and Bacteroidetes [Bacteroidota] [28–30], reduced levels of *Clostridium* and the overall Firmicutes [Bacillota] phylum [29,31,32], and variations in *Bacteroides* and *Prevotella* based on the consumed diet [33–36]. The composition and diversity of the gut microbiota are thought to play a significant role in weight regulation [37]. Specifically, a higher Firmicutes to Bacteroidetes ratio (F/B), referring to the relative abundance or proportion of the two major phyla of bacteria in the human gut microbiota, has been linked to obesity [38], although there is no consensus on the use of this ratio [39,40]. Ethnicity greatly influences microbiota composition and diversity [41,42], making it crucial to conduct ethnic-specific research. In this context, we embarked on a prospective study to explore the effect of SG on energy homeostasis, gut peptides, hormones, and gut microbiota in a sample of Emiratis with obesity. We aim to understand the unique mechanisms relevant to weight loss following SG, beyond the restrictive effect of the surgery on food intake. Through an in-depth exploration of these mechanisms, we aspire to lay the groundwork for formulating less invasive anti-obesogenic treatment strategies.

## Materials and methods

As illustrated in the supporting information (S1 Flow Chart).

### Study design

The study design involved a three-month prospective, pre-post investigation.

We recruited Emiratis with obesity undergoing SG from two hospitals in the UAE, one in Sharjah and one in Dubai, between October 27, 2019, and March 26, 2021.

The study was registered in ClinicalTrials.gov (ID: NCT04200521)

### Sample size calculation

The sample size was determined by the F/B ratio, which is the principal consequence of this research. By employing traditional calculations based on normal distributions and the findings of Damms-Machado et al., in which the mean (SD) fecal F/B changed significantly from 5.9 (2.1) to 10.4 (1.4) in the three months after bariatric surgery [29], it was determined that a sample size of two patients would be sufficient to achieve 80% power at a two-sided 5% significance level to identify an effect of comparable magnitude. Considering the anticipated high dropout rate, the minimal sample size was multiplied by 15. Thus, we targeted thirty adult Emiratis with obesity of both sexes and resided in the UAE intending to undergo SG. A total of 43 eligible participants were recruited; however, only 19 of them underwent SG and were subsequently included in the study.

### Informed consent

#### Information for participants.

We provided each participant with an information sheet that detailed the purpose, duration, procedures, and protocols involved, potential risks and benefits, and their rights as participants, including the right to withdraw at any time without penalty.

#### Consent process.

The consent process involved training the research staff, explaining the study in detail, answering any questions, and ensuring that participants fully understood the information provided. We conducted this interaction in the participants’ preferred language to ensure their comprehension.

#### Documentation.

Participants who agreed to take part in the study signed a written informed consent form. We securely stored these forms to ensure confidentiality and adhere to ethical standards.

### Ethical considerations

The Dubai Health Care Regulatory Research Ethics Committee (DHCR-REC), the Research Ethics Committee of the Ministry of Health and Prevention of the UAE (MOHP/DXB-REC-52/2018), and the Zayed University Ethical Committee Board (ZU19_51_F) granted ethical approvals for the study. Before enrollment, written informed consent was obtained from all patients. In studies involving human subjects, every procedure was conducted in adherence to the ethical principles set forth by the relevant national and institutional research committees, in addition to the 1964 Helsinki Declaration.

### Inclusivity in global research

Additional information regarding the ethical, cultural, and scientific considerations specific to inclusivity in global research is included in the Supporting Information (S1 Checklist).

### Inclusion criteria

Patients determined to undergo sleeve gastrectomy (SG) by the multidisciplinary team at the partner hospitals were evaluated for their eligibility for the study by the research team. Inclusion criteria entailed Emirati participants residing in the UAE, with stable body weight for the past three months and free of antibiotics for at least three months, aged between 18 to 60 years, of either sex, with a body mass index (BMI) of ≥ 35 kg/m^2^, and consenting to participate in the study.

### Exclusion criteria

The study excluded individuals who were either pregnant or nursing during that period.

Additionally, we excluded individuals who had used antibiotics within the previous three months due to potential alterations in gut microbiota composition that could influence study results. We also excluded participants who experienced significant weight loss of 5% or more in the three months before surgery, as this could potentially impact baseline metabolic parameters.

Moreover, individuals with excessive alcohol consumption, defined as exceeding two standard drinks per day for males and one standard drink per day for females, were excluded from the study [43].

### Study variables

#### General demographics and medical history.

The subsequent information was gathered: age, sex, comorbidities (including hypertension, dyslipidemia, diabetes mellitus type 2 (T2DM), and cardiovascular disease), surgical history within the previous five years, weight loss of at least 5% over the previous three months, present medication usage (including antibiotic consumption), prebiotic and probiotic supplement consumption, nutritional supplement intake, and level of physical activity.

We analyzed physical activity levels using two questions from the initial screening questionnaire. “Do you integrate physical activity into your daily routine?” and “Do you incorporate any exercise program?” required a “yes” or “no” response. Sedentary was defined as “no” to both questions, light physical activity as “yes” to the first question, moderate physical activity as “yes” to the second question, and high physical activity as “yes” to both.

#### Nutritional assessment.

**Anthropometrics:** The participant’s body weight was determined to the nearest kilogram using a portable Seca 762 scale (Vogel & Halke, Hamburg, Germany) while fasting and wearing lightweight apparel, and removing their footwear. Height was determined to the nearest millimeter using a portable Seca stadiometer while they were shoeless. BMI was calculated as weight (kg)/ height (m^2^). Using measuring tapes (Seca 201- Ergonomic circumference measuring tape), the waist circumference (WC) was determined at the midpoint, halfway between the right iliac crest and the lower costal region, with an accuracy of 0.1 cm [44]. The WC was then, classified and categorized as appropriate or increased based on the cut-off points of < 94 cm and ≥ 94 cm for males and < 80 and ≥ 80 cm for females, respectively [45]. Waist-to-height ratio (WHtR) was determined by dividing WC by height in centimeters; abdominal obesity was defined as a WHtR value of 0.5; a WHtR value below 0.5 was regarded as normal [46].

**Body composition:** Percentage body fat (PBF), fat mass (FM), and fat-free mass (FFM) were evaluated using a bioelectrical impedance analyzer (BC-420 MA, Tanita Corporation, Tokyo, Japan), following the manufacturer’s instructions. We defined elevated PBF among males and females using cut-offs of ≥  25% and ≥  35%, respectively [47].

**Resting energy expenditure (REE):** Resting energy expenditure was assessed using a portable indirect calorimeter (Metabolic Analyser - Indirect Calorimetry; Breezing, Halberstadt, Germany). We conducted this measurement under resting conditions, requiring participants to refrain from eating or drinking for a minimum of 2 hours before the analysis.

Before each measurement, the breezing device was calibrated according to the manufacturer’s instructions. Calibration involved setting up the device to accurately measure oxygen (VO2) and carbon dioxide (VCO2) levels. Participants were instructed to wear a mouthpiece provided with the calorimeter. A nose clip was utilized to ensure that participants breathed exclusively through the mouth, minimizing the risk of air leakage and optimizing measurement accuracy. Participants stayed in a comfortable position to minimize movement. The device continuously records gas exchange data during a designated period of normal breathing (3 minutes). Finally, the collected data is analyzed by the device’s software to determine participants’ REE by analyzing the oxygen and carbon dioxide content of breath. This process provides a reliable assessment of their resting metabolic rate [48].

**Food intake:** Was assessed using three 24-hour dietary recalls, during which the patient described and quantified the foods and beverages over the past 24 hours for three consecutive days, including two weekdays and one weekend day. During the interview, the dietitian probed the participants more than once to cover forgotten eating occasions and foods and assisted the participant in estimating portion size to provide comprehensive information about the intake. Daily energy (Kcal) and fiber intakes (g/1000 Kcal) were calculated using the food composition database of the Nutritics software (using the Middle Eastern version of their software program).

**Appetite:** The assessment was conducted using the Simplified Nutritional Appetite Questionnaire (SNAQ) [49]. SNAQ is a quick and easy-to-administer self-assessment screening tool composed of four items (1, 2, 4, and 6) of the Council on Nutrition Appetite Questionnaire (CNAQ). These items assess appetite, satiety, the taste of food, and the number of meals per day, whereby each question has five letter answer options ranging from a to e, which respectively stand for a = 1; b = 2; c = 3; d = 4, and e = 5. The sum of these items constitutes the SNAQ score, which ranges from 4 to 20, with higher scores indicating better appetite and a lower risk of malnutrition. A cut-off of fourteen indicates a risk of malnutrition and predicts a significant risk of at least 5% weight loss within six months.

We translated the questionnaire into Arabic according to international procedures [50].

Two independent translators first translated it both forward and backward, compared the two versions, and made necessary adjustments to the Arabic version.

**Postoperative bariatric diet meal planning (**S1 File**):** Participants start with clear liquids (Stage 1) and advance to full liquids (Stage 2) within several days post-surgery, ensuring at least 60 ounces (1,800 cc) of fluid intake daily to avoid dehydration. They then progress through pureed foods (Stage 3), soft foods (Stage 4), and finally all solids (Stage 5) throughout 4 to 6 weeks, with typical daily caloric intake increasing from 400 kcal/day initially to 1,200–1,500 kcal/day several months post-surgery [51].

### Biochemical evaluation

#### Gut peptides (total GLP-1, GLP-2, PYY, CCK, GHR) and hormones (insulin and leptin).

A total of 3 vacutainers of ethylenediaminetetraacetic acid (EDTA) tubes of venous blood samples were collected after a 12-hour overnight fast by a registered nurse/phlebotomist using a 22-gauge needle. We immediately stored the collected blood in EDTA-coated tubes on ice and centrifuged it at 3000 rpm for 15 minutes at a refrigerated temperature. Then, plasma was collected and stored at −20 ºC for later analysis at the Research Institute for Medical and Health Sciences (RIMHS), Sharjah University. Total GLP-1, GLP-2, PYY, GHR, insulin, and leptin were measured using sandwich-based enzyme-linked immunosorbent assay (ELISA) kits (Diametra Millipore, St. Louis, Missouri, USA). We measured CCK using a competitive ELISA kit from ABclonal Technology (Wuhan, China), adhering to the manufacturer’s instructions. The minimum detectable concentrations of these assays are 1.5 pm; 0.3 ng/ml; 6.5 pg/mL; 50 pg/mL; 2 µ U/mL; 0.2 ng/mL, 4.93 pg/ml for GLP-1, GLP-2, PYY, GHR, insulin, leptin, and CCK, respectively.

The measurement of fasting plasma glucose (FPG) levels was conducted following the manufacturer’s instructions using a portable Lux Metre Blood Test (Biochemical Systems International, S.p.A.; Arezzo, Italy). In addition, the Homeostatic Model Assessment of Insulin Resistance (HOMA-IR) was computed by multiplying fasting serum insulin (mU/mL) by FPG (mg/dL) and dividing the result by 405 [52]. HOMA-IR values exceeding 1.8 were regarded as suggestive of insulin resistance [53].

### Gut microbiota

#### Stool sample collection.

Stool samples were self-collected into Zymo (DNA/RNA) Shield™ fecal collection tubes and stored at room temperature without refrigeration. From each participant, two samples were obtained: one was subjected to analysis, and the other one was retained to verify accuracy. Collection of samples adhered to established protocols. To collect 1 gram of feces, a spoon was affixed to the cap of each collection tube, which was pre-filled with 9 ml of DNA/RNA Shield™. We collected sixty-two stool samples from the 43 eligible subjects three days pre-surgery and from the 19 included participants three months post-operatively. The analysis included paired samples from the nineteen participants who underwent SG.

#### DNA isolation and sequencing of amplicons of the V3–V4 region of the 16S rRNA gene.

The DNA isolation and sequencing of the barcoded amplicons of the V3-V4 region of the 16S rRNA gene were performed by the established protocols supplied by Illumina (Illumina, Eindhoven, The Netherlands) [54]. The steps taken were as follows: the sequencing procedure was executed utilizing the Illumina MiSeq system (San Diego, CA, USA), employing barcodes, and the 2x300 bp protocol. The software Quantitative Insights Into Microbial Ecology (QIIME 2) (https://qiime2.org) software [55] was utilized to analyze the raw sequences. The latter were classified utilizing the Silva database (version 132) as the reference database for the 16S rRNA gene.

We filtered the reads and took only those included in at least 20% of the samples. We use the old nomenclature for phyla, providing new names in brackets in the text for proper comparison with previous literature.

#### Diversity analysis.

**α Diversity indices:** The following diversity indices (Observed Operational Taxonomic Units (OTUs), Chao1 index, Phylogenetic diversity (Faith’s PD), Pielou’s evenness, and Shannon diversity index) were calculated with QIIME 2 and displayed in R Studio using the R software (4.1.x) (R Core Team, http://www.R-project.org): We created boxplots using the ggplot2 package for R. We set the depth of rarefication at 3800.

**β Diversity indices:** Principal Coordinate Analysis (PCoA) was performed using QIIME2 software to visualize dissimilarities in β-diversity. The analysis utilized Unweighted UniFrac, Weighted UniFrac, Bray-Curtis dissimilarity, and Jaccard similarity matrices and were converted to 2D-plots in R using the phyloseq package. Eigenvalues were computed to determine the variance explained by each principal coordinate.

We used Permutational Multivariate Analysis of Variance (PERMANOVA) [56] to assess the statistically significant differences in β diversity between the two groups.

When there were more than two categories, we used the non-parametric Kruskal-Wallis method to calculate the p-value in pairwise comparisons for the diversity between each category.

The non-parametric Wilcoxon signed-rank test for paired samples corrected with the Benjamini–Hochberg false discovery rate (FDR) for multiple comparisons was used in R to analyze differences in microbiota in participants pre- and post-surgery (at phylum and genus levels). The composition of the two groups was visualized using the Krona tool (https://github.com/marbl/Krona/wiki/KronaTools). We calculated the F/B ratio by dividing the relative abundances of Firmicutes and Bacteroidetes.

We investigated the microbial taxa with differential abundances between pre- and post-surgery using linear discriminant analysis (LDA) effect size (LEfSe) (version 1.0 http://huttenhower.sph.harvard.edu/galaxy/). Taxa in each group were deemed enriched if their absolute log10 LDA scores were greater than 2.

A Spearman correlation analysis, for non-parametric data, as is usual for microbiota analyses, was carried out between genera and continuous variables (BMI, WC, WHtR, PBF, REE, energy intake, fiber intake/1000 kcal, appetite, insulin, leptin, GLP1, GLP2, PYY, CCK, and GHR) to pinpoint the microorganisms that correlated positively or negatively with surgical results. The Spearman rho ranges between values of | 0.20 | and | 0.39 | ; | 0.4 | and | 0.69 | ; | 0.70 | and | 0.89; | 0.90 | and | 1.00 | denote a weak, moderate, strong, and very strong correlation, respectively [57]. At a cut-off of < 0.05 for q-values (adjusted p-values after the False Discovery Rate (FDR) correction), were considered significantly different. We generated a Spearman correlation analysis heatmap in R software using the corrplot package.

### Statistical analysis

The general characteristics of the participants were analyzed utilizing version 21 of the Statistical Package for the Social Sciences software (SPSS Inc. Chicago, IL, USA). The normality of the data was assessed using the Shapiro-Wilk test before selecting the appropriate statistical analyses. The Shapiro-Wilk test evaluates the null hypothesis (H₀) that the data follows a normal distribution. A P-value greater than 0.05 indicates that we fail to reject the null hypothesis, suggesting that the data does not significantly deviate from normality and can be considered normally distributed (weight (W = 0.94; p = 0.32), BMI (W = 0.95; p = 0.44), WC (W = 0.96; p = 0.58), WHtR (W = 0.97; p = 0.84), fat mass (FM) (W = 0.9; p = 0.06), fat-free mass (FFM) (W = 0.92; p = 0.15), PBF (W = 0.96; p = 0.61), REE (W = 0.95; p = 0.48), EI (W = 0.95; p = 0.44), Fiber intake (W = 0.94; p = 0.29), Fiber/1000Kcal (W = 0.94; p = 0.27), appetite score (W = 0.94; p = 0.32), HOMA-IR (W = 0.92; p = 0.14), GLP1 (W = 0.95; p = 0.54), GLP2 (W = 0.94; p = 0.3), CCK (W = 0.92; p = 0.15), leptin (W = 0.92; p = 0.16), insulin (W = 0.95; p = 0.52), and GHR (W = 0.94; p = 0.36)). Conversely, a P-value less than 0.05 means we reject the null hypothesis, indicating that the data significantly deviates from normality and should be considered non-normally distributed (FPG (W = 0.54; p < 0.001) and PYY (W = 0.9; p = 0.001)).

Continuous variables are presented as mean ±  standard error (SE) for normally distributed data and as median with interquartile range (IQR) for non-normally distributed data. Within-subject changes after bariatric surgery were evaluated by a paired sample T-test for normally distributed continuous variables, the Wilcoxon signed-rank test (NPT) for non- normally distributed data, and the McNemar test for categorical variables. A two-tailed p-value less than 0.05 was deemed to indicate statistical significance.

## Results

### Effects of bariatric surgery on anthropometrics and plasma levels of gut hormones

The baseline characteristics of the participants are detailed in Table 1 along with the study variables comparing the conditions before and after bariatric surgery.

**Table 1:** Demographics, anthropometrics, and biochemical parameters between pre- and post-bariatric surgery (n = 19).

Variable	Pre-surgery	Post-surgery	Std. error mean and 95%confidence interval of the difference (CI lower, upper %)	p-value	Statistical test (t/Z)
Age (years) (M ±SE)	29.78 ± 2.2	**–**	**–**	**–**	**–**
Sex n (%)					
Female	12 (63.2)	**–**	**–**	**–**	**–**
Male	7 (36.8)	**–**	**–**	**–**	**–**
**Weight (kg) (**M ± SE)	120.33 ± 7.37	98.38 ± 6.57	1.3 (19.21, 24.67)	<0.001^a^	t = 16.87
**Height (cm)** (M ± SE)	166 ± 2.31	–	–	–	–
**BMI** (kg/m^2^) (M ± SE)	43 ± 1.98 (10)	35 ± 1.83	0.38 (6.98, 8.59)	<0.001^a^	t = 20.36
**WC (cm) (**M ± SE)	126 ± 4.84	111 ± 4.94	1.46 (12, 18)	<0.001^a^	t = 9.97
**WHtR (**M ± SE)	0.75 ± 0.02	0.66 ± 0.02	0.009 (0.06, 0.10)	<0.001^a^	t = 9.53
**FM (kg) (**M ± SE)	54 ± 3.92	38 ± 3.58	1.65 (12.5, 19.5)	<0.001^a^	t = 9.69
**FFM (kg) (**M ± SE)	61 ±2.83	56 ± 2.64	0.99 (3.3, 7.5)	<0.001^a^	t = 5.43
**PBF (%) (**M ± SE)	46 ± 1.42	40 ± 1.86	1.22 (4, 9)	<0.001^a^	t = 5.44
**REE (Kcal) (**M ± SE)	1679 ± 82.96	1805 ± 53.92	−75.1 (−284, 32)	0.110	t = −1.68
**Energy Intake (kcal) (**M ± SE)	2454 ± 171.7	756 ± 67.6	155.27 (1372, 2024)	<0.001^a^	t = 10.93
**Fiber Intake (g) (**M ± SE)	12 ± 1.43	5.9 ± 0.86	0.85 (4.2, 7.8)	<0.001^a^	t = 7.04
**Fiber g/ 1000 Kcal (**M ± SE)	5 ± 0.51	7.5 ± 0.71	0.71 (−4.1, −1.1)	0.002^a^	t = −3.65
**Appetite (**M ± SE)	16.58 ±0.37	11.74 ± 0.5	0.61 (3.5, 6.1)	<0.001^a^	t = 7.83
**FPG (mg/dl) (**Mdn (IQR))	98 (12.88)	98.5 (16.25)	–	0.896	Z = −0.13
**HOMA-IR** (M ± SE)	7 ± 1.12	2.5 ± 0.5	0.75 (2.9, 6.06)	<0.001^a^	t = 5.91
**Insulin (mU/mL) (**M ± SE)	25 ± 2.91	9 ± 1.59	2.32 (11.01, 20.8)	<0.001^a^	t = 6.83
**Leptin (ng/mL) (**M ± SE)	45 ± 2.77	28 ± 2.91	1.57 (13.54, 20.14)	<0.001^a^	t = 10.71
**GLP1 (pM) (**M ± SE)	25 ± 2.67	12 ± 1.23	2.27 (8.18, 17.74)	<0.001^a^	t = 5.7
**GLP2 (ng/mL) (**M ± SE)	2.6 ± 0.24	1.96 ± 0.2	0.123 (0.36, 0.88)	<0.001^a^	t = 5.07
**PYY (pg/mL) (**Mdn (IQR))	48 (17.01)	116 (22.1)		<0.001^a^	Z = −3.54
**CCK (pg/mL) (**M ± SE)	69.5 ± 6.71	54.86 ± 3.38	6.07 (2, 27.5)	0.026^a^	t = 2.42
**GHR (pg/mL) (**M ± SE)	568 ± 15.01	693 ± 41.98	42.26 (−215, −37)	0.008 ^a^	t = −2.97

^a^Denotes statistical significance between the two groups (p < 0.05); The statistical significance was evaluated by Paired sample T-test for normally distributed continuous variables (t test); Wilcoxon signed-rank test (NPT) for non-normally distributed variables (Z test). BMI: Body mass index, WC: Waist circumference, WHtR: Waist-to-height ratio, FM: Fat mass, FFM: Fat-free mass, PBF: Percentage body fat, REE: Resting energy expenditure, FPG: Fasting plasma glucose; HOMA-IR: Homeostatic model assessment of insulin resistance, GLP-1, GLP-2: Glucagon-like peptides 1 and 2, PYY: Peptide YY, CCK: Cholecystokinin, GHR: ghrelin.

Ultimately, 19 subjects were included pre- and post-surgery: mean age of 29.78 ±  2.29; included 7 males (36.8%) and 12 females (63.2%). Among the nineteen participants enrolled in the study, n =  1 had hypertension, and n =  1 had dyslipidemia; however, none were diagnosed with T2DM or cardiovascular disease. Regarding surgical history, two participants had undergone cholecystectomy, one had a history of appendectomy, and one had a prior caesarean section. Preoperative medication regimens included one participant being prescribed antihypertensive medication, another receiving antacid therapy, and a third utilizing antihistamine medication. Additionally, one participant was taking multivitamins before surgery. Following surgery, the majority of participants received multivitamins and iron supplements, while one continued antacid therapy, another continued antihypertensive medication, and a third continued antihistamine therapy. Notably, none of the participants reported the use of prebiotics, probiotics, or fiber supplements either before or after the surgical intervention. There were no significant differences in the level of physical activity observed between the pre- and post-surgery groups. Furthermore, no one consumed alcohol, and there was no difference in smoking habits. (S1 Table). Additionally, none of the study participants had postoperative complications. Three months post-surgery, the mean BMI fell from 43 to 35 kg/m^2^ (t = 20.36; p < 0.001); similarly, waist circumference (t = 9.97; p < 0.001), waist-to-height ratio (t = 9.53; p < 0.001), and PBF (t = 5.44; p < 0.001) showed significant improvements. Appetite reduced significantly post-surgery. Participants reduced their total daily energy intake from (245 4*Kcal*± 171) pre-surgery to (75 6*Kcal*± 67.6) (t = 10.93; p < 0.001) at three months post-surgery. We noted no significant differences regarding REE and FPG.

Post-operatively, the HOMA-IR displayed a significant reduction; however, it remained above 1.8. Moreover, fasting levels of insulin (t = 6.83; p < 0.001), leptin (t = 10.71; p < 0.001), GLP-1 (t = 5.7; p < 0.001), GLP-2 (t = 5.07; P < 0.001), and CCK (t = 2.42; P = 0.026) decreased significantly, and GHR (t = −2.97; p = 0.008) and PYY (Z = −3.54; p < 0.001) levels significantly increased.

### α- and β-diversity (microbial diversity and richness, microbial dissimilarities)

#### α-Diversity.

**Fig 1:**
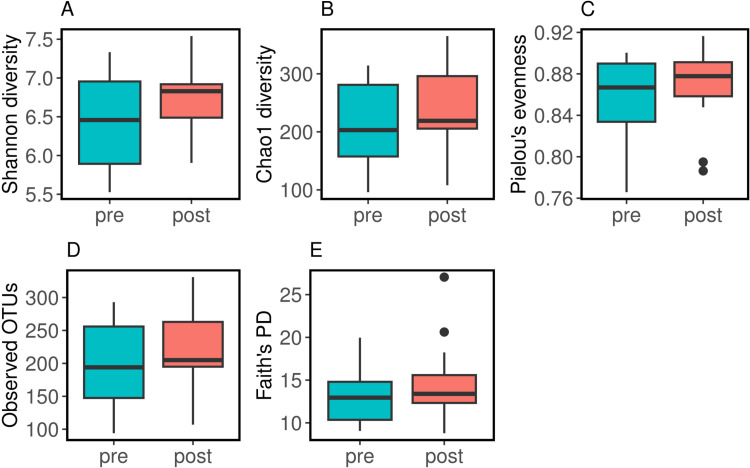
α-diversity pre- and post-SG-surgery. (A) Shannon; (B) Chao1; (C) Pielou’s evenness; (D) Observed OTUs; (E) Faith’s PD.

The α-diversity of the pre-and post-surgery samples (Fig 1) was calculated using Kruskall-Wallis for five different metrics, including Shannon diversity (Fig 1A; pre (Mean (SEM) =  6.47 ±  0.14, post =  6.73 ±  0.10; p = 0.21), Chao1 (Fig 1B; pre =  213.2 ±  16.14, post =  241.16 ±  16.83; p = 0.23), Pielou’s evenness (Fig 1C; pre =  0.85 ±  0.009, post =  0.87 ±  0.008; p = 0.31), observed OTUs (Fig 1D; pre =  199.31 ±  14.7, post =  222.05 ±  13.74; p = 0.28) and Faith’s PD (Fig 1E, pre =  13.2 ±  0.71, post =  14.58 ±  0.93; p = 0.28). We noted no statistically significant differences in the five-diversity metrics between pre- and post-surgery.

#### β-diversity.

**Fig 2:**
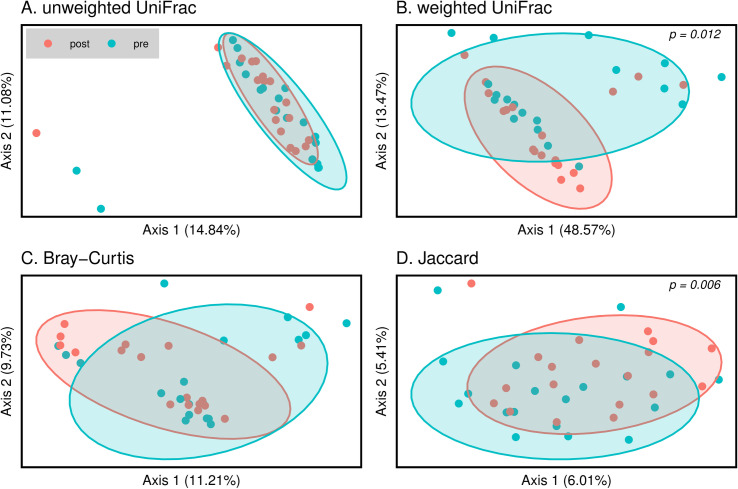
Principal Coordinate Analysis (PCoA) based on (A) Unweighted UniFrac, (B) Weighted UniFrac, (C) Bray-Curtis dissimilarity, and (D) Jaccard similarity. The total variance explained by the first two components is displayed on the PC1 and PC2 axes, respectively. Eigenvalues for the first five axes (PC1 to PC5) are provided in S3 Table. Pre-surgery (blue spheres) and post-surgery (red spheres).

Regardingβ-diversity (Fig 2), no significant differences were observed for Unweighted UniFrac and Bray-Curtis dissimilarity metrics. However, significant differences were identified for Weighted UniFrac and Jaccard metrics between pre-and post-surgery ((Fig 2B; p = 0.012; (Eigenheim-values PC1: 0.80, PC2: 0.22 and Fig 2D; p = 0.006; Eigenheim-values PC1: 0.82, PC2: 0.74, respectively). All p values were calculated with the statistic test PERMANOVA.

Pseudo-F for PERMANOVA for the different analyses were as follows: Unweighted UniFrac: F = 1.960337; Weighted Uni: F = 1.691676; Bray: F = 1.272821 and Jacc: F = 1.314021. Eigenheim values for the first five principal coordinates are detailed in S3 Table.

### Relative abundance at the phylum level

Bacteroidetes [Bacteroidota], Firmicutes [Bacillota], and Proteobacteria [Pseudomonadota] dominated the microbiome composition of obese Emirati participants pre- and post-surgery (S1A and S1B Fig). The detailed proportions of the top 7 phyla in the pre-and post-surgery samples are provided in (Table 2). Proteobacteria [Pseudomonadota] and Saccharibacteria [Saccharimonadota] exhibited a statistically significant increase in their proportion post-surgery (Z = −1.9; q = 0.013 and Z = −2.4; q = 0.027, respectively). No changes were noted regarding the relative abundance of Firmicutes and Bacteroidetes nor the F/B ratio between pre- and post-surgery samples.

**Table 2:** Comparison of gut microbial composition at the phylum level pre- and post-surgery.

Phylum	Pre-surgery (Mdn (IQR))	Post-surgery (Mdn (IQR))	q-value	Statistical test (Z)
**Firmicutes** **[Bacillota]**	0.49 (0.16)	0.44 (0.13)	0.3	−0.88
**Bacteroidetes [Bacteroidota]**	0.46 (0.15)	0.47 (0.15)	0.7	−0.12
**Proteobacteria [Pseudomonadota]**	0.028 (0.017)	0.04 (0.025)	0.013^a^	−1.9
**Actinobacteria [Actinomycetota]**	0.005 (0.006)	0.005 (0.007)	0.65	−0.08
**Lentisphaerae** **[Lentisphaerota]**	0 (0)	0 (0)	0.3	−0.88
**Fusobacteria [Fusobacteriota]**	0 (0)	0 (0.0001)	0.1	−1.7
**Saccharibacteria [Saccharimonadota]**	0 (0.00008) (	0.0001 (0.0004)	0.027^a^	−2.4
**F/B ratio**	1.14 (0.65)	0.95 (0.65)	0.084	−0.4

^a^q-value equal to or less than 0.05 was considered statistically significant for Wilcoxon Signed-Rank test with FDR correction.

Mdn (IQR), median (interquartile range).

### Relative abundance at the genus level

**Fig 3:**
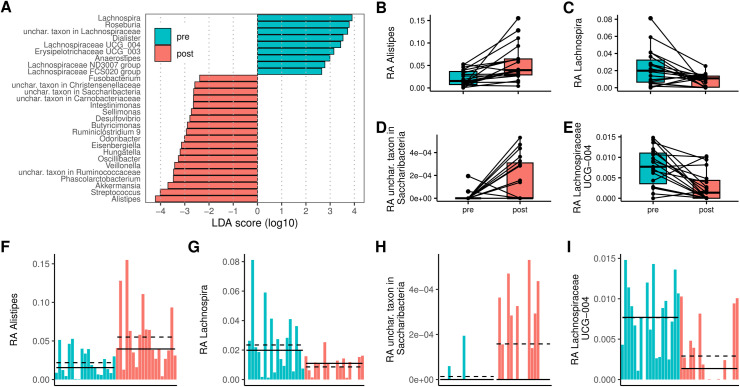
Boxplots display the median (as the central tendency) and the interquartile range (25th to 75th percentiles) as the bottom and top edges of the box. The whiskers extend to the most extreme data points not considered outliers (1.5 times the interquartile range), and the outliers are plotted individually. (A) LefSe discriminative analysis between pre- and obese post-surgery samples (absolute LDA score >  2). The LDA score (log10) for the more prevalent genera in the pre-surgery is represented on a positive scale (green), and the LDA score for the more prevalent genera in the post-surgery is defined on a negative scale (red). (B) Paired boxplots of Alistipes in pre- and post-surgery samples. (C) Paired boxplots of Lachnospira in pre- and post-surgery samples. (D) Paired boxplots of Saccharibacteria in pre- and post-surgery samples. (E) Paired boxplots of Lachnospiraceae UCG 004 in pre- and post-surgery samples. (F) Relative abundance of Alistipes in pre- and post-surgery samples. Means (straight line) and medians (dotted line) are shown for both groups. (G) Relative abundance of Lachnospira in pre- and post-surgery samples. Means (straight line) and medians (dotted line) are shown for both groups. (H) Relative abundance of Saccharibacteria in pre- and post-surgery samples. Means (straight line) and medians (dotted line) are shown for both groups. (I) Relative abundance of Lachnospiraceae UCG 004 in pre- and post-surgery samples. Means (straight line) and medians (dotted line) are shown for both groups.

A total of 28 genera showed differences in abundance between pre- and post-surgery with absolute LDA scores >  2, using LefSe (Fig 3A). In this case, of the twenty-eight genera, the same five genera significantly decreased post-surgery as for the Wilcoxon signed-rank test (Table 3 and Fig 3B–I), while 11 genera that were significantly increased by the Wilcoxon signed-rank test also increased post-surgery as identified by LEfSe.

**Table 3:** Changes in gut microbial taxa (Genus level)pre- and post-surgery.

Genus	Pre-surgery (Mdn (IQR))	Post-surgery (Mdn (IQR))	q-value	Statistical test (Z)
**Decreased in post-surgery**				
***Lachnospiraceae* UCG-004**	0.007 (0.008)	0.001 (0.004)	0.001^a^^,^^b^	−3.18
***Lachnospira***	0.02 (0.03)	0.01 (0.01)	0.027^a^^,^^b^	−2.45
***Erysipelotrichaceae* UCG-003**	0.0008 (0.004)	0 (0)	0.021^a^^,^^b^	−2.62
***Dialister***	0.0086 (0.014)	0.0006 (0.008)	0.038^a^^,^^b^	−2.33
***Lachnospiraceae* FCS020 group**	0.0007 (0.001)	0 (0.00008)	0.049^a^^,^^b^	−2.27
***Lachnospiraceae***	0.027 (0.024)	0.019 (0.013)	0.056^b^	−2.17
***Lachnospiraceae* ND3007 group**	0.0005 (0.001)	0 (0.0007)	0.06^b^	−2.13
***Roseburia***	0.022 (0.026)	0.012 (0.018)	0.05^b^	−2.21
***Anaerostipes***	0.004 (0.005)	0.001 (0.003)	0.07^b^	−2.07
**Increased in post-surgery**				
***Streptococcus***	0.001 (0.001)	0.013 (0.013)	0.000009^a^^,^^b^	−3.82
***Alistipes***	0.015 (0.03)	0.039 (0.038)	0.0006^a^^,^^b^	−3.34
***Desulfovibrio***	0 (0)	0.0004 (0.003)	0.006^a^^,^^b^	−3.06
***Carnobacteriaceae***	0 (0)	0.0003 (0.0006)	0.008^a^^,^^b^	−2.97
***Eisenbergiella***	0 (0)	0.0005 (0.003)	0.009^a^^,^^b^	−2.93
***Hungatella***	0 (0)	0.0007 (0.002)	0.009^a^^,^^b^	−2.93
**Uncharacterized genus in *Ruminococcaceae***	0.002 (0.001)	0.005 (0.005)	0.009^a^^,^^b^	−2.89
***Sellimonas***	0 (0)	0 (0.001)	0.02^a^^,^^b^	−2.66
***Butyricimonas***	0.0005 (0.002)	0.002 (0.003)	0.027^a^^,^^b^	−2.53
***Saccharibacteria***	0 (0)	0 (0.0003)	0.031^a^^,^^b^	−2.49
***Oscillibacter***	0.0017 (0.004)	0.004 (0.007)	0.038^a^^,^^b^	−2.33
***Fusobacterium***	0 (0)	0 (0.0002)	0.21^b^	−1.54
***Intestinimonas***	0 (0.0005)	0.0005 (0.0015)	0.05^b^	−2.27
**Uncharacterized genus in *Christensenellaceae***	0 (0.00008)	0.00009 (0.0009)	0.08^b^	−1.99
***Ruminiclostridium* 9**	0.0017 (0.004)	0.0035 (0.005)	0.05^b^	−2.21
***Odoribacter***	0.0035 (0.005)	0.007 (0.006)	0.06^b^	−2.09
***Veillonella***	0.0006 (0.0016)	0.003 (0.007)	0.06^b^	−2.13
***Phascolarctobacterium***	0.0005 (0.009)	0.008 (0.017)	0.06^b^	−2.11
***Akkermansia***	0 (0.0002)	0.0008 (0.015)	0.11^b^	−1.87
*Clostridiales*	0 (0)	0 (0.0003)	0.043^a^	−2.38
*Haemophilus*	0 (0.0009)	0.0005 (0.01)	0.049^a^	−2.29
Uncharacterized genus in *Coriobacteriaceae*	0 (0)	0 (0.0005)	0.049^a^	−2.31

^a^q-value equal to or less than 0.05 was considered statistically significant with FDR correction. The non-parametric Wilcoxon signed-rank test was used.

^b^Taxa that were different as identified by LEfSe with an LDA effect score >2.

Changes noted at the level of the following genera were only statistically significant using LEfSe, including *(Lachnospiraceae* and *Lachnospiraceae* ND3007 group*, Roseburia, Anaerostipes* (pre-surgery); *Fusobacterium, Intestinimonas,* an uncharacterized genus in *Christensenellaceae, Ruminiclostridium* 9*, Odoribacter, Veillonella, Phascolarctobacterium, Akkermansia* (post-surgery).

Using the non-parametric Wilcoxon signed-rank test (for paired samples), 19 genera significantly changed between pre-and post-surgery, as depicted in (Table 3 and Fig 3B–I). Of the 19 genera, five decreased post-operatively (*Lachnospiraceae* UCG-004, *Lachnospira, Erysipelotrichaceae* UCG-003, *Dialister,* and *Lachnospiraceae* FCS020 group), while the remaining 14 increased post-surgery.

### Correlations between microbial taxa and clinical parameters

The taxa that show statistically significant correlations with clinical parameters are provided in the S2 Table and S2 Fig, along with the correlation coefficients (rho).

Several taxa that significantly increased post-surgery (uncharacterized genera in *Ruminococcaceae*, *Desulfovibrio*, *Akkermansia, Carnobacteriaceae*, *Streptococcus, and Sellimona*s) were negatively correlated with energy intake, BMI, and appetite. *Roseburia*, which decreased post-surgery, was positively correlated with appetite, while PYY was positively correlated with genera that increased post-surgery (*Carnobacteriaceae*, *Streptococcus, Eisenbergiella, Hungatella, Veillonella*) and negatively correlated with *Dialister* that decreased post-surgery. Lachnoclostridium and Flavonifractor showed a negative correlation with leptin. Hungetella and Eisenbergiella, which were significantly higher post-surgery, showed a positive correlation with GHR.

There were no observed correlations among the gut microbiota at the genus level and the subsequent anthropometric and dietary variables (WC, WHtR, PBF) REE, fiber/ 1000 kcal; or biochemical variables (insulin, GLP1, GLP2, and CCK).

## Discussion

There is a growing body of evidence linking the gut microbiota to obesity; however, the impact of bariatric surgery on the gut microbiota and the mechanism of action is understudied. Moreover, the integrated communication between the gut microbiota and the metabolic and neuroendocrine systems, along with its connection to obesity, remains to be uncovered. Some of the significant determinants of gut microbiota composition are geography, ethnicity, and cultural practices; thus, gut bacteria composition must be studied in several areas of the globe. We explored the impact of bariatric surgery on the gut microbiota and the factors that influence it, such as energy balance, gut peptides, and hormones, in a sample of Emiratis with obesity. Participants achieved significant weight loss post-surgery, with significant differences in the gut microbiota composition.

Obesity is associated with dysbiosis and less bacterial α-diversity. Post-surgery, our sample experiences an increase in α-diversity without reaching statistical significance. This was consistent with the literature, where patients’ post-surgery, α-diversity, and metabolic profiles improved [58]. However, the published data on the matter is inconsistent. While some research indicated a significant increase [59–63], others found no change or even a decrease [32,42,58,64] post-surgery on gut bacteria. According to Palleja et al. [65], Roux-en-Y gastric bypass leads to an increase in microbial diversity, which remains stable even one year postoperatively. Another comparative study between RYGB and sleeve gastrectomy (SG) also reported greater microbial diversity in the RYGB group, whereas no significant changes were observed in microbial diversity before and after SG in obese individuals [66]. The discrepancies may be the result of many factors, such as the type of surgery conducted [58,66], the sample characteristics at baseline, and the diets followed post-surgery. Therefore, changes in α-diversity appear not to be a reliable indicator of the effectiveness of bariatric surgery in humans [67]. Additionally, the alterations in gut microbiota following RYGB, including specific taxonomic shifts (e.g., greater abundances of Escherichia, Bacteroides, and Veillonella), may play a role in the differing metabolic outcomes between the two procedures [68].

Most literature pinpoints a major change in the microbial community after bariatric surgery, suggesting that the latter significantly impacts the gut microbiota, facilitates weight loss, and ameliorates metabolic profile [59,67]. We observed significant changes in β-diversity post-surgery, denoting a heterogeneous microbiota [69]. However, not all four metrics were significant, with the weighted UniFrac (which considers abundance) being significant, but also Jaccard similarity (which does not take abundance into account). Therefore, we also looked at which taxa were differentially abundant after SG surgery.

We observed significant differences in the relative abundance of several bacterial taxa. In pre-and post-surgery, Bacteroidetes and Firmicutes dominated the microbiome composition, with Proteobacteria the third most phylum, but trailing by an order of magnitude. The other phyla were even less abundant. Post-surgery, Proteobacteria, and Saccharibacteria significantly increased. The rise in Proteobacteria is consistent with earlier findings [30,63,70–72]. Increases in luminal acidity and dissolved oxygen following SG favor the growth of these microbes [30]. A rise in Proteobacteria after bariatric surgery is associated with favorable outcomes, including weight loss [73], enhanced glucose homeostasis, and reduced systemic inflammation [74], even though this phylum is also known to harbor (potential) pathogenic species.

Despite some previous reports that obesity is associated with a higher F/B ratio that decreases after surgery [18,75], F/B remained constant post-surgery in our study. F/B may not be associated with weight loss or predict the outcome post-surgery [39,72,76]. Instead, obesity may be caused by an enhanced energy harvest from one’s diet, not just by a simple rise in F/B ratio but also by gut bacteria tuned towards creating SCFA. Therefore, looking into the connection between energy harvest associated with bacterial SCFA synthesis and obesity is warranted [77]. In line with the literature, we observed significant decreases in several genera, including *Lachnospiraceae* [78]*, Dialister*, *Roseburia*, *Anaeostipes, and Eryspelotrichaceae* [18]*.* Noteworthy, increased appetite positively correlated with *Roseburia* in our study. This is in line with previous findings showing that this genus predominated in the microbiota pre-surgery and decreased post-surgery which could explain the decrease in appetite noted post-surgery [18,71]. Furthermore, bariatric surgery induced an increase in the relative abundance of *Butyricimonas,* which has been linked to decreased appetite [79]. As expected, *Akkermansia*, which is usually low in obesity, increased post-surgery [72] approximately 6-fold (not significant in the Wilcoxon test, but an LDA of −3.5 in LEfSe). The mucin-degrading properties of *Akkermansia* reduce inflammation and alterations in adipose tissue, and this bacterium has been strongly linked to favorable metabolic outcomes and a decline in body weight [67,80]. There is a significant correlation between an increase in *Akkermansia abundance* and a reduction in the taste of energy-dense foods [32]. This is consistent with the hypothesis that *Akkermansia* prefers an environment with lower food intake, as in our context, where it negatively correlates with energy intake. *Akkermansia* enrichment is a potential therapy for obesity management. Indeed, a randomized clinical trial showed that short-term oral administration of pasteurized *Akkermensia* to overweight and obese participants led to weight loss and improved insulin sensitivity [81].

Several studies [18,71] reported increased *Alistipes* (phylum Bacteroidetes), *Veilonella*, and *Streptococcus* post-surgery. We found similar results. Increases in these strains are usually associated with metabolic improvements and more significant weight loss [18,71]. Furthermore, we found a positive correlation between PYY (the satiety hormone) and *Veilonella* and *Streptococcus* in our study. Specifically, greater abundances of *Veillonella* and *Streptococcus* have been associated with T2DM remission following Roux-en-Y gastric bypass (RYGB) [82].

The gut-brain axis (GBA), which controls hunger and food intake, and the intestinal microbiota consist of bidirectional communication [83]. Some bacterial strains can affect the production of gut hormones, such as PYY, GLP-1, leptin, and GHR, that control hunger and satiety in the brain [83,84] and regulate energy and glucose homeostasis [75,85]. Most evidence refers to increased GLP-1 post-surgery [86]. Interestingly, we observed a decrease in fasting GLP-1, as also shown in some studies [75,87], suggesting that enhanced insulin sensitivity may lead to the downregulation of GLP-1. Methodological differences might explain this discrepancy, i.e., assessing fasting post-prandial GLP-1 response vs. testing a metabolite or the active form of GLP-1. Therefore, it remains challenging to draw firm conclusions about GLP-1 post-SG [87]. Our participants also showed increased plasma PYY post-surgery, as demonstrated elsewhere [24,75,85], partially attributed to weight loss and enhanced satiety. PYY is a neuropeptide that has satiating and reducing effects on body weight and adiposity [88]. Conversely, GHR promotes food intake and attenuates the anorectic effect of PYY and GLP-1 [89]. Gastric cells primarily. Removing part of the stomach may lower GHR, as shown in most SG studies [85]. Yet, we unexpectedly found that GHR increased. A possible explanation is that the majority of GHR-producing cells are located in the stomach’s fundus, whereas circulating acyl GHR may come primarily from the duodenum, which remains intact in SG, leading to a compensatory upregulation of duodenal GHR production [88]. In GHR-deficient mice, SG was similarly effective in inducing weight loss, raising the question of whether this hormone is a crucial facilitator of weight reduction [87], especially since weight loss post-surgery is independent of GHR [90].

The considerable reduction in insulin levels in our study is one of the most significant outcomes of bariatric surgery [91]. Similarly, post-surgery, there was a significant decrease in HOMA-IR levels [92]. Enhanced insulin sensitivity promotes weight loss and decreased appetite [90]. Similarly, leptin resistance improved post-surgery. Leptin stimulates anorexigenic neurons and reduces appetite [84]. As discussed above, changes in the colonic microbiome post-surgery correlate with variations in appetite. Sanmiguel et al. [32] found a link between having higher levels of certain types of colonic bacterial species and losing your appetite after SG. These bacteria included *Odoribacter.* We report a similar finding, where appetite also dropped significantly post-surgery, associated with an increase in *Roseburia* and *Butyricimonas*. Future studies should explore causal processes linking the effects of increasing these bacteria on appetite.

Energy intake, BMI, and appetite negatively correlated with several taxa that significantly increased post-surgery were with. Among them were *Streptococcus, Akkermansia*, a genus in *Carnobacteriaceae,* and a genus in *Ruminococcaceae.* It has been demonstrated that dietary fiber affects hunger, body weight, and gut microbiota composition [93]. Participants consumed more fiber/1000 kcal post-SG, which could play a role. However, they did not meet the recommended daily level. Interestingly, although we detected several significant gut microbiota correlations with clinical parameters, none were reported for resting energy expenditure [94,95].

The question of whether the changes noticed post-bariatric surgery are permanent or reversible remains debatable and potentially surgery-specific [96]. The most extensive modifications in the composition of the gut microbiota are usually present post-RYGB [42]. SG induces less drastic alterations in the diversity of the adaptive gut microbiota than RYGB because it does not alter the gut’s anatomical structure [ 42,79]. Apart from the type of surgical intervention [30,97], the inconsistencies in the impact of bariatric surgery on the gut microbiota are partly due to the differences in participant characteristics, such as geographic location [98], ethnicity, sex, age, initial BMI, initial microbial composition, gastrointestinal comorbidities, medication use, prebiotics/probiotics use, differences in diet, as well as study design, sampling time, sample storage method, sequencing methodology, bioinformatics and statistical software used [42], all of which can change gut microbial composition.

### Strengths and limitations

To the best of our knowledge, this is the only study that has examined the effect of bariatric surgery on the following gut peptides (GHR, GLP-1, GLP-2, CCK, PYY) and hormones including insulin, and leptin, on Emiratis with obesity post-SG-surgery. Furthermore, this study’s strengths include a novel research question targeting ethnic-specific research, a clear data-extraction process, well-defined inclusion and exclusion criteria, and a wide range of parameters assessed through valid methods. On the other hand, our results are limited by the small number of patients and the limited follow-up period, although small samples are common in such studies [29,33,97,99]. Furthermore, changes in these subjects’ gut microbiota are evident. According to studies using several follow-up time points, most microbiome alterations occur within three months post-surgery, followed by a steady microbiome for the next year [100]. We did not measure the post-prandial secretion of gut hormones in response to a standardized meal test. Additionally, other gut hormones such as the appetite reducing GIP-oxyntomodulin hybrid peptide were not measured, which could provide further insights into metabolic changes post-surgery [101]. Another limitation is that we did not account for diet, a flaw that is present in the majority, if not all, of human bariatric studies. Diet following bariatric surgery may alter significantly over time, which could impact the gut microbiota and should be investigated. Furthermore, our study did not include other bariatric surgery techniques, such as Roux-en-Y gastric bypass (RYGB), limiting the opportunity to investigate consistency or potential variations between diverse bariatric surgery procedures [34]. Despite the observational study design’s inability to establish causality for changes reported post-surgery, it allows for the formulation of several hypotheses. Another limitation of this study is the potential lack of generalizability of the findings. The study exclusively focused on the Emirati population, which was chosen due to the scarcity of gut microbiota data in the GCC region and the unique ethnic influences that could impact microbiota composition. Although this population provides valuable insights into how gut microbiota and bariatric surgery outcomes interact in a largely understudied region, the results may not be directly applicable to other ethnic or regional populations with different genetic, dietary, and lifestyle factors. Future studies are recommended to explore these associations across diverse ethnic groups and geographic regions to enhance the generalizability of the findings and to determine if region-specific factors or genetic predispositions contribute to the observed effects.

### Practical implications

Understanding changes post-bariatric surgery in gut peptides, hormones, and gut microbiota and the interplay between these factors can lead to non-invasive, anti-obesogenic personalized interventions, including targeted probiotics, prebiotics, dietary, and pharmacological interventions. Based on our results, we suggest interventions to reduce *Roseburia* levels and/or increase *Butrycimonas*, ultimately influencing appetite. Furthermore, the administration of probiotics rich in *Akkermensia* to treat obesity appears promising and warrants empirical research.

## Conclusion

We discovered that bariatric surgery significantly alters the plasma levels of gut peptides and hormones, as well as the composition of the gut microbiota. We speculate that the microbiota modifications could, at least in part, be responsible for the positive outcomes of bariatric surgery, including weight loss. Most importantly, we could not confirm the association between obesity and an increased F/B ratio [102,103]. Further research is necessary to understand the interplay between the gut microbiota and changes in gut peptides, hormones, human metabolism, and energy homeostasis following bariatric surgery.

## Supporting information

S1 Flow Chart Overview of the study process. (DOCX)

S1 Fig Krona plot of the intestinal bacterial communities pre (A) and post-surgery (B) at the phylum level. The _ in both plots indicates “uncharacterized.”
(PDF)

S2 Fig Heatmap of Spearman correlation analysis between gut microbiota (at genus level) and anthropometrics and clinical variables. The asterisk (*) denotes statistical significance at a cut-off of < 0.05 for q-values (adjusted p-values after the False Discovery Rate (FDR) correction) and the negative and positive values are the rho ( = correlation factor), which can be positive for positive correlations and negative for negative correlations.
(PDF)

S1 Table Lifestyle factors for pre-and post-bariatric surgery. (DOCX)

S2 Table Spearman correlation coefficient (rho) for the taxa showing significant negative and positive correlations with clinical data according to the heatmap (S2 Fig). (DOCX)

S3 Table Eigenvalues of principal coordinates (PCs) for different beta diversity metrics. (DOCX)

S1 File Postoperative bariatric diet meal planning. (DOC)

S1 Checklist Inclusivity in global research. (DOCX)
