# Supplementary material for: Changes in energy homeostasis, gut peptides, and gut microbiota in Emiratis with obesity after bariatric surgery
Source: PLoS One. 2025 Feb 24;20(2):e0318699. doi: 10.1371/journal.pone.0318699 (PMC11849869; doi:10.1371/journal.pone.0318699)
Supplement: S1 Flow Chart — (DOCX) [file pone.0318699.s001.docx]

**S1 Flow chart. Overview of the study process**

19 Emirati adults scheduled to undergo sleeve gastrectomy (BMI≥35 kg/m2) from 2 hospitals

| Training of Research Assistants on Data Collection and training of Participants on the method of stool collection  Baseline assessment (pre-surgery) and Follow Up Assessment (3 months Post Bariatric Surgery) on all study parameters   - 24-hour diet recall (EI) - Indirect calorimetry (REE) - Appetite questionnaire ^*^ - Anthropometrics **(**BMI, WC, WHtR, FM, FFM and % BF) - Plasma Gut Peptides & Hormones (measured via ELISA): PYY, GLP-1&2, GHR, CCK, insulin, leptin - Gut Microbiota Composition ( Phylum and genus-level analysis, (F/B) ratio, α and β diversity   Main Results   - Decreased energy intake and appetite - Decreased BMI, waist circumference, and body fat - No significant differences regarding REE and FPG - Increased PYY and GHR levels - Decreased insulin, leptin and GLP-1 levels - No significant changes in Shannon, Chao1, Pielou's evenness, observed OTUs, or Faith's PD post-surgery. - Significant change in β-diversity - Changes in phylum and genus-level composition - F/B ratio remained constant - Negative correlation of EI, BMI, and appetite with several increased taxa post surgery     **Abbreviations:** BMI: Body mass index, EI: Energy intake, REE: Resting energy expenditure,  FPG: Fasting plasma glucose, WC: Waist circumference,WHtR: Waist-to-height ratio, FM: Fat mass,  FFM: Fat-free mass, PBF: Percentage body fat, PYY: Peptide YY, GLP-1, GLP-2: Glucagon-like peptides, 1 and 2,  GHR: ghrelin, CCK: Cholecystokinin, F/B: Firmicutes/ Bacteroidetes ratio. |  |  |  |  |  |  |  |  |  |  |  |  |  |  |  |  |  |  |  |  |  |  |  |  |  |  |  |
| --- | --- | --- | --- | --- | --- | --- | --- | --- | --- | --- | --- | --- | --- | --- | --- | --- | --- | --- | --- | --- | --- | --- | --- | --- | --- | --- | --- |
