# Supplementary figures and images for: Changes in energy homeostasis, gut peptides, and gut microbiota in Emiratis with obesity after bariatric surgery
Source: PLoS One. 2025 Feb 24;20(2):e0318699. doi: 10.1371/journal.pone.0318699 (PMC11849869; doi:10.1371/journal.pone.0318699)

S1A Fig.

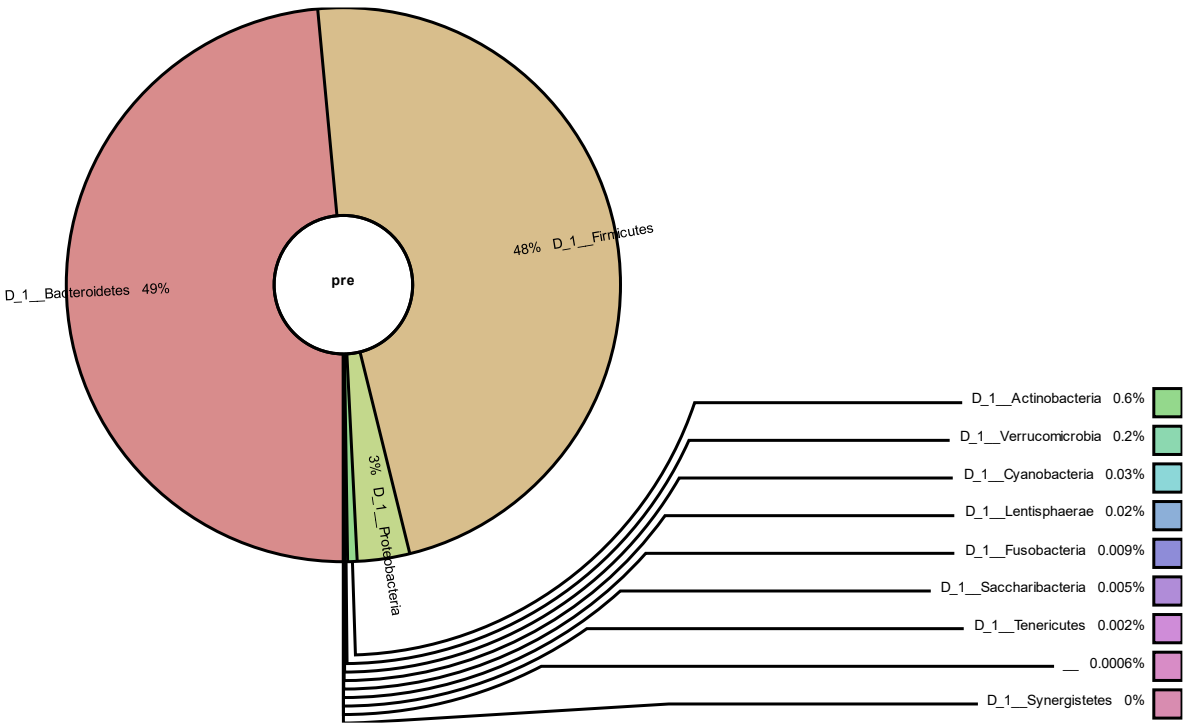

S1B Fig.

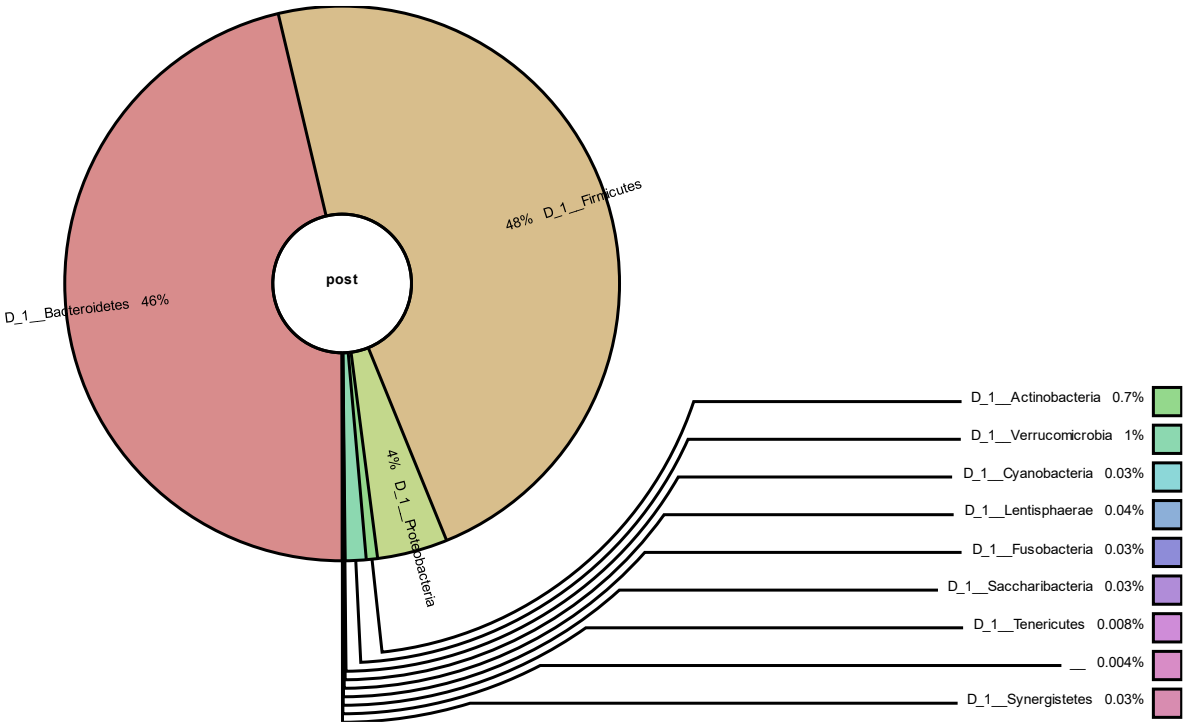

Supplement: S1 Fig — The _ in both plots indicates “uncharacterized.” (PDF) [file pone.0318699.s002.pdf]

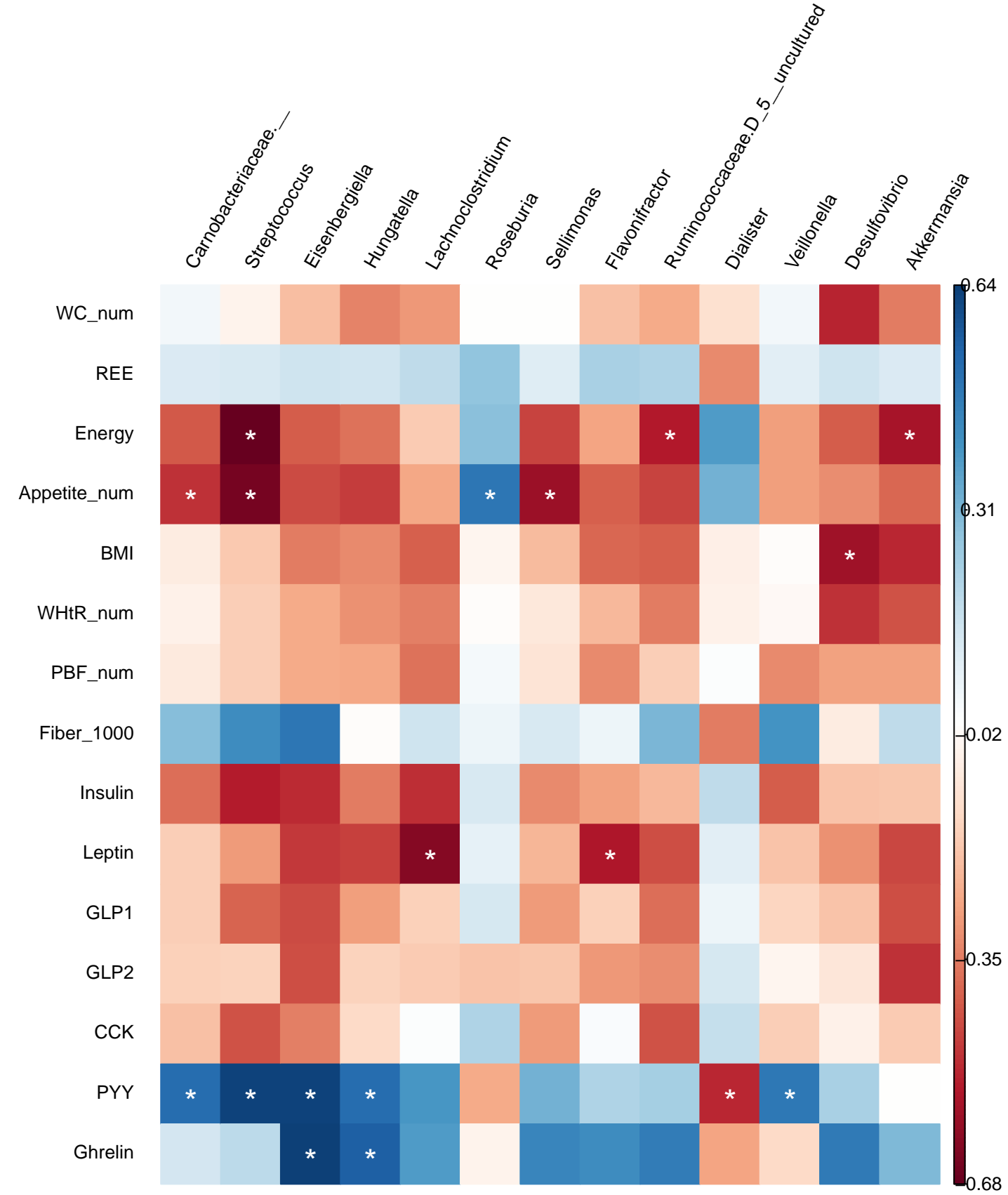

Supplement: S2 Fig — The asterisk (*) denotes statistical significance at a cut-off of < 0.05 for q-values (adjusted p-values after the False Discovery Rate (FDR) correction) and the negative and positive values are the rho ( = correlation factor), which can be positive for positive correlations and negative for negative correlations. (PDF) [file pone.0318699.s003.pdf]
