# Supplementary material for: Changes in energy homeostasis, gut peptides, and gut microbiota in Emiratis with obesity after bariatric surgery
Source: PLoS One. 2025 Feb 24;20(2):e0318699. doi: 10.1371/journal.pone.0318699 (PMC11849869; doi:10.1371/journal.pone.0318699)
Supplement: S1 Table — (DOCX) [file pone.0318699.s004.docx]

**S 1 Table. Lifestyle factors for pre- and post-bariatric surgery (n=19).**

| **Lifestyle factors** | **Pre-surgery** | **Post-Surgery** | **p-value** |
| --- | --- | --- | --- |
| **Probiotic Supplement - Yes, n (%)** | 0 (0) | 0 (0) | - |
| **Fiber Supplement - Yes, n (%)** | 0 (0) | 0 (0) | - |
| **Prebiotic Supplement - Yes, n (%)** | 0 (0) | 0 (0) | - |
| **Medication use - Yes, n (%)** | 3 (15.8) | 3 (15.8) | 1 |
| **Nutritional Supplements -Yes n (%)** | 1 (5.3) | 16 (84.2) | <0.001^a^ |
| **Smoking -Yes n (%)** | 3 (15.8) | 4 (21.1) | 0.33 |
| **Alcohol consumption- Yes n (%)** | 0 (0) | 0 (0) | - |
| **Physical activity level** |  |  | 0.163 |
| **Sedentary** | 2 (10.5) | 3 (15.8) |  |
| **Light** | 16 (84.2) | 11 (57.9) |  |
| **Moderate** | 1 (5.3) | 1 (5.3) |  |
| **High** | 0 (0) | 4 (21.1) |  |

^a^Denotes statistical significance between the two groups (p<0.05); The statistical significance was evaluated for categorical variables by the McNemar test.
