# Supplementary material for: Changes in energy homeostasis, gut peptides, and gut microbiota in Emiratis with obesity after bariatric surgery
Source: PLoS One. 2025 Feb 24;20(2):e0318699. doi: 10.1371/journal.pone.0318699 (PMC11849869; doi:10.1371/journal.pone.0318699)
Supplement: S2 Table — (DOCX) [file pone.0318699.s005.docx]

**S2 Table. Spearman correlation coefficient (rho) for the taxa showing correlations with clinical data according to the heatmap (S2 Fig).**

| **BMI** | | **EI** | **Appetite** | **Leptin** | **GHR** | **PYY** |
| --- | --- | --- | --- | --- | --- | --- |
| **Genus** |  |  |  |  |  |  |
| **An uncharacterized taxon within *Carnobacteriaceae*** | - | - | -0.49 | - | **-** | +0.52 |
| ***Streptococcus*** | - | -0.68 | -0.64 | - | **-** | +0.62 |
| ***Eisenbergiella*** | - | - | - | - | +0.6 | +0.62 |
| ***Hungatella*** | - | - | - | - | +0.55 | +0.52 |
| ***Lachnoclostriduum*** | - | - | - | -0.62 | **-** | - |
| ***Roseburia*** | - | - | +0.49 | - | **-** | - |
| ***Sellimonas*** | - | - | -0.58 | - | **-** | - |
| ***Flavonifractor*** | - | - | - | -0.54 | **-** | - |
| **An uncharacterized taxon within *Ruminococcaceae*** | - | -0.54 | - | - | **-** | - |
| ***Dialister*** | - | - | - | - | **-** | -0.51 |
| ***Veillonella*** | - | - | - | - | **-** | +0.48 |
| ***Desulfovibrio*** | -0.57 | - | - | - | **-** | - |
| ***Akkermansia*** | - | -0.56 | - | - | **-** | - |

The negative and positive Spearman rho ranges between values of |0.20| and |0.39|; |0.4| and |0.69|; |0.70| and |0.89; |0.90| and |1.00| denote a weak, moderate, strong, and very strong correlation, respectively. Only statistically significant correlations are presented in the table at a cut-off of <0.05 for q-values (adjusted p-values after the False Discovery Rate (FDR) correction).
