# Supplementary material for: Changes in energy homeostasis, gut peptides, and gut microbiota in Emiratis with obesity after bariatric surgery
Source: PLoS One. 2025 Feb 24;20(2):e0318699. doi: 10.1371/journal.pone.0318699 (PMC11849869; doi:10.1371/journal.pone.0318699)
Supplement: S3 Table — (DOCX) [file pone.0318699.s006.docx]

**Supplementary Table 3** Eigenvalues of Principal Coordinates (PCs) for Different Beta Diversity Metrics

| **Beta Diversity Metric** | **PC1 Eigenvalue** | **PC2 Eigenvalue** | **PC3 Eigenvalue** | **PC4 Eigenvalue** | **PC5 Eigenvalue** |
| --- | --- | --- | --- | --- | --- |
| Unweighted UniFrac | 0.7899 | 0.5899 | 0.5899 | 0.5899 | 0.5899 |
| Weighted UniFrac | 0.7997 | 0.2218 | 0.2218 | 0.2218 | 0.2218 |
| Bray-Curtis | 1.3166 | 1.1431 | 1.1431 | 1.1431 | 1.1431 |
| Jaccard | 0.8177 | 0.7357 | 0.7357 | 0.7357 | 0.7357 |
