# Supplementary material for: Changes in energy homeostasis, gut peptides, and gut microbiota in Emiratis with obesity after bariatric surgery
Source: PLoS One. 2025 Feb 24;20(2):e0318699. doi: 10.1371/journal.pone.0318699 (PMC11849869; doi:10.1371/journal.pone.0318699)
Supplement: S1 File — (DOC) [file pone.0318699.s007.doc]

**S1 File: Postoperative Bariatric Diet Meal Planning (Mechanick et al., 2019)**

**Stage 1: Bariatric Clear Liquids (1 to 3 days after surgery)**

 Provide six to eight small feedings of clear liquid foods. Begin with sips of water, and then add bouillon or clear broth, unsweetened juices (apple, cranberry, or grape juices), diet gelatin, and herbal tea like anise, or chamomile. Generally, it should be low in calories and sugar and free of caffeine, carbonation, and alcohol.

 If tolerated well, progress to Bariatric Full Liquids

**Stage 2: Bariatric Full Liquids (10 to 14 days after surgery)**

 Provide six to eight small feedings per day. Begin with high protein liquids/shakes/ soups or specialized high protein (low fat, low sugar-free, high protein drinks).

The combined volume of the six to eight small feedings plus water intake should be at least 48 to 64 oz/day (6‐8 cups) to meet hydration needs.

**Stage 3: Bariatric Pureed Diet (10 to 14 days)**

If tolerated well, advance to a pureed diet (stage 3).

-Puree diet is food blended or liquefied with adequate fluid, eaten without chewing, and the consistency and texture should progress gradually.

 Provide four to six small feedings of pureed foods. Use high-quality protein foods such as scrambled eggs, low-fat or cottage cheese, or blenderized lean meats such as tuna, fish, and chicken. Strained baby foods are a convenient option. Integrate a high-quality protein food with each meal or snack.

 Portion: 2 to 4 oz (4 to 8 tbsp) at a time of solid foods.

 Consume protein food first, vegetables and fruit second, and starch foods last to help ensure adequate protein consumption.

 Alternate fluid intake with food intake. Avoid consuming fluids with meals. Wait at least 30 minutes after consumption of solid foods or meals to drink fluids.

 Avoid chewing gum. If swallowed, gum can block the stomach opening.

 Avoid drinking from straws because the air swallowed can cause bloating and stretch the pouch.

 Behavior techniques must be applied and reinforced (e.g., eating small amounts, eating slowly, and chewing food thoroughly before swallowing).

**Stage 4: Bariatric soft Diet (1 to 2 weeks)**

 If intake is improving, advance to soft foods.

Textured-modified: To limit stage-4 foods to those that can be mashed or do not require excessive chewing (chopped or ground foods).

 Behavior techniques must be applied and reinforced (e.g., eating small amounts, eating slowly, and chewing food thoroughly before swallowing).

***Stage 5:*Bariatric Diet (regular consistency)**

After 1 or 2 weeks on soft foods, start introducing some solid food and progress to all solids as tolerated (stage 5), generally 4 to 6 weeks after surgery.

 Provide four to six small feedings of regular consistency food. Use high-quality protein foods such as scrambled egg, low-fat cheese or cottage cheese, or lean meats such as tuna fish, chicken,

 Alternate fluid intake with food intake. Consume fluids at least 30 minutes before or after solid foods.

 Should keep a food record to document food intake and eating behavior, including foods tolerated or not tolerated, to discuss with the dietitian during follow-up visits.

**If patients do not progress through these stages of their diet in the appropriate periods due to nausea, vomiting, or dysphagia, careful evaluation of nutrition should be performed, and the surgeon should consider investigating potential causes**

Mechanick, J. I., Apovian, C., Brethauer, S., Garvey, W. T., Joffe, A. M., Kim, J., . . . Still, C. D. (2019). CLINICAL PRACTICE GUIDELINES FOR THE PERIOPERATIVE NUTRITION, METABOLIC, AND NONSURGICAL SUPPORT OF PATIENTS UNDERGOING BARIATRIC PROCEDURES - 2019 UPDATE: COSPONSORED BY AMERICAN ASSOCIATION OF CLINICAL ENDOCRINOLOGISTS/AMERICAN COLLEGE OF ENDOCRINOLOGY, THE OBESITY SOCIETY, AMERICAN SOCIETY FOR METABOLIC & BARIATRIC SURGERY, OBESITY MEDICINE ASSOCIATION, AND AMERICAN SOCIETY OF ANESTHESIOLOGISTS - EXECUTIVE SUMMARY*. Endocr Pract,* 25(12), 1346-1359. doi:10.4158/gl-2019-0406
